# Visceral Leishmaniasis (Kala-Azar): A Triumph Against a Trickster Disease

**DOI:** 10.7759/cureus.25698

**Published:** 2022-06-06

**Authors:** Fajar Pasha, Shoaib Saleem, Talha Nazir, Javera Tariq, Khadija Qureshi

**Affiliations:** 1 Internal Medicine, Rawalpindi Medical University, Rawalpindi, PAK; 2 Internal Medicine, Holy Family Hospital, Rawalpindi, PAK; 3 Internal Medicine, Mayo Hospital, Lahore, PAK; 4 Neurology, AINeuroCare Academy, Dallas, USA; 5 Department of Pathology, Pakistan Institute of Medical Sciences, Islamabad, PAK; 6 Internal Medicine, Bucks County Kidney Specialists, Langhorne, USA

**Keywords:** kala-azar, visceral leishmaniasis, amastigotes, febrile infant, pancytopenia

## Abstract

Leishmaniasis, a protozoan parasitic pathology, is caused by infection with a parasite known as *Leishmania donovani*, which is transmitted to humans through the bite of a sand fly. The disease has various manifestations, including cutaneous leishmaniasis, visceral leishmaniasis (VL), and disseminated cutaneous leishmaniasis. Visceral leishmaniasis (VL), also known as kala-azar, is mostly seen in the Asian and African regions and can be deadly if left untreated. We present the case of a 10-month-old male infant who was brought to the office with the chief complaints of recurrent fever for the past one month, along with generalized fatigue and poor feeding. A comprehensive history, detailed physical examination, and laboratory testing, including bone marrow aspiration, were performed, and visceral leishmaniasis was diagnosed.

## Introduction

Leishmaniasis is a disease of the tropical and subtropical regions caused by an intracellular parasite, *Leishmania*. The bite of *Phlebotomus orientalis *(sand fly) causes its transmission to humans. This infectious pathology is found in the vast majority of tropics across all continents. However, Northeastern Africa, Southern Europe, the Middle East, Southeastern Mexico, Central and South America, and South Asia show a higher case incidence. A species of subgenus *Leishmania* is *Leishmania donovani*, which develops mainly in the midgut and foregut of *Phlebotomus orientalis* (sand fly) [1]. It is endemic in the Asian and African regions, with a higher disease burden in Afghanistan, Pakistan, Syria, Saudi Arabia, Algeria, Iran, Brazil, and Peru in the case of cutaneous leishmaniasis. The cases of visceral leishmaniasis (VL) are seen mainly in India, Bangladesh, Nepal, Sudan, and Brazil [2].

Although direct transmission is by the bite of infected sand flies, human-to-human transmission (mediated by vectors), laboratory accidents, and transmission through domestic animals (serving as alternate reservoirs) have also been documented in some endemic areas. Risk factors for leishmaniasis include being in the endemic regions, genetic susceptibility, low immunocompetence, poor nutrition, and other comorbidities [1].

Based on clinical presentation, leishmaniasis has three main forms: cutaneous (localized and disseminated), mucocutaneous, and visceral or kala-azar. Visceral leishmaniasis has an incubation period of 3-8 months. Presenting signs and symptoms include prolonged fever, weight loss, fatigue, pallor, hepatosplenomegaly, lymphadenopathy, pancytopenia, and skin pigmentation. Treatment options mainly include pentavalent antimonials, amphotericin B, pentamidine, lipid forms of miltefosine, and paromomycin [2].

## Case presentation

A 10-month-old male infant was brought to the office by his parents. The patient had been suffering from recurrent fever of low to moderate degrees ranging from 100.8°F to 102°F for the past month. In addition, there were irregular bouts of fever associated with rigors and chills. The fever was temporarily reduced by over-the-counter acetaminophen. The parents also reported that the child was not feeding well and appeared visibly pale and fatigued. Vital signs were recorded as blood pressure of 90/65 mm Hg, heart rate of 130 beats per minute (bpm), temperature of 101°F, and respiratory rate of 24 breaths per minute. The child lived in a small village near the woods in Northern Pakistan and was the fourth born of a consanguineous marriage. He was delivered via normal spontaneous vaginal delivery at home. Birth history was insignificant, and the infant had not received any vaccinations because of parental health beliefs. He was exclusively breastfed until his weaning at four months of age. He had a regular dietary intake until a month ago when his febrile symptoms appeared. The family had a poor socioeconomic background with malnutrition in their other children.

General physical examination showed a crying, emaciated infant in moderate distress. There were numerous petechiae on the forearms and thighs. The child weighed 24 pounds (lbs), and his height was measured at 29.5 inches, giving a body mass index (BMI) of 19.4 kg/m^2^. No previous medical records were available to compare the growth charts, but the parents reported an adequate developmental history with normal acquisition of age-appropriate milestones. Abdominal examination revealed a protruding, non-tender abdomen with palpable hepatosplenomegaly. No bruits were heard on auscultation, and bowel sounds were normal. Several enlarged lymph nodes were noted in the cervical, axillary, and groin regions. A review of all other systems was within the normal range. Complete blood counts (CBCs), liver function tests (LFTs), thyroid function tests (TFTs), basic metabolic profile (BMP), clotting profile, and a chest X-ray were ordered. Abdominal ultrasonography confirmed hepatosplenomegaly. The CBC results were deranged, showing moderate normocytic anemia with a hemoglobin of 7.6 g/dL, a leukocyte count of 3.6 × 10^9^/L, and a low platelet count of 45 × 10⁹/L. The results are tabulated below (Table 1).

**Table 1:** Laboratory results for blood cell counts.

Parameter	Finding	Reference range
Hemoglobin (Hb)	7.6 g/dL	11.3-14.1 g/dL
Hematocrit	30%	32%-41%
Mean cell volume	78 fL	81-94 fL
Leukocytes	3.6 × 10⁹/L	4-10 × 10⁹/L
Platelets	45 × 10⁹/L	140-340 × 10⁹/L

**Figure 1:**
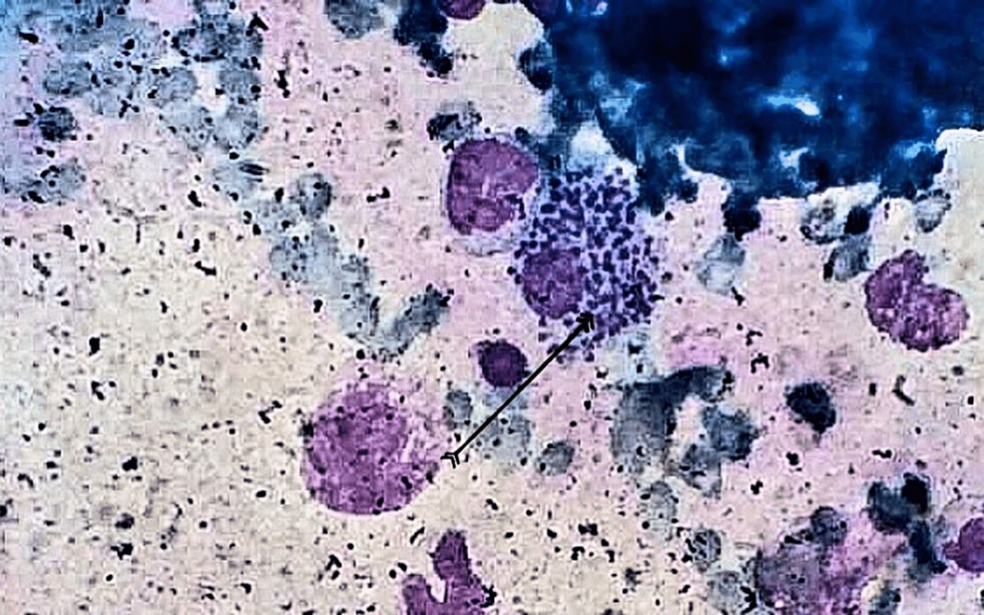
Microscopic analysis of bone marrow aspirate showing intracellular amastigotes within macrophages (black arrow).

Given the history and the presenting complaints, the patient was tested to rule out malaria, typhoid, infectious mononucleosis, and tuberculosis. The negative results of the differential diagnoses and pancytopenia prompted a bone marrow biopsy. Microscopic analysis of the bone marrow aspirate revealed several Leishman-Donovan (LD) bodies. Numerous intracellular and extracellular amastigotes were seen in the aspirate, thus confirming the diagnosis of visceral leishmaniasis or kala-azar. The bone marrow findings are shown below (Figure 1).

Treatment was initiated immediately with liposomal amphotericin B and acetaminophen. The patient was started on formula feeds to ensure proper nourishment. He responded well to therapy and showed a significant improvement over the next 2-3 weeks. He was followed up monthly for the next six months. The parents were extensively counseled regarding nutrition, vaccination, and the importance of regular health visits.

## Discussion

Leishmaniasis is an infectious pathology caused by a vector-borne, obligate intracellular, protozoan parasite called *Leishmania*. Leishmaniasis is a complex disease with diverse clinical manifestations, including mucocutaneous, cutaneous (disseminated and localized), and visceral leishmaniasis [3]. Worldwide, approximately 350 million people are at risk of contracting the disease. Leishmaniasis has an incidence rate of 1.5-2 million cases annually, with a mortality rate of 70,000 deaths per year [1]. Visceral leishmaniasis (VL) cases are estimated to be around 100,000 per year, with more than 95% of cases reported from Brazil, China, Ethiopia, India, Kenya, Nepal, Somalia, and Sudan. Risk factors for leishmaniasis include poverty, population migration, malnutrition, poor hygiene, and an immunocompromised state [3].

The *Leishmania* parasite has been categorized in more than 20 subspecies, transmitted by approximately 70 different types of phlebotomine sand flies. Sand flies are found on all continents except Oceania. They are nocturnal in habit, and the prey is usually oblivious of the fly bite. *Leishmania donovani* is the main causative agent of visceral leishmaniasis (VL) in South Asia (Bangladesh, India, Pakistan, and Nepal) and East Africa (Sudan, Somalia, Ethiopia, and Kenya) [1-3].

The female *Phlebotomus orientalis* (sand fly) carrying the *Leishmania donovani* serves as the vector and transmits *Leishmania* to humans or other animal reservoirs. As per Ethiopian studies, *Leishmania donovani* infects the sand fly when there is an approximate parasite load of 20,000 per mL of blood in the host. Usually, more than half of reservoirs remain asymptomatic. The *Leishmania donovani* species has an anthroponotic transmission, meaning it can be transmitted from human to human without any mediating reservoir [1]. Its life cycle consists of two phases, the initial phase being the promastigote, which later develops into the amastigote. The promastigotes possess flagella for motility inside the sand fly’s intestines. Upon biting the host, the sand fly injects the promastigote into the skin, where it enters the bloodstream. The host macrophages phagocytose the promastigotes, which then become the amastigotes, also known as the Leishman-Donovan body. The growing and multiplying amastigotes within the blood and lymph nodes trigger the immune system, causing the asymptomatic or symptomatic disease [4].

The most deadly form of leishmaniasis is visceral disease. The species associated with visceral leishmaniasis (VL) include *Leishmania donovani*, *Leishmania infantum*, and *Leishmania chagasi* [5]. It causes a systemic infection affecting the liver, spleen, and hematogenous and reticuloendothelial system. Visceral leishmaniasis predominantly targets infants and children as their developing immune systems can be susceptible. However, adults are usually unaffected or asymptomatic because of acquired immunity [3].

The signs and symptoms of visceral leishmaniasis include pancytopenia, fatigue, weight loss, hepatosplenomegaly, and disseminated intravascular coagulation. It also leads to a grayish discoloration of the skin (hence the name black fever/kala-azar). Anemia is usually the most common hematological manifestation. After being phagocytosed into the mononuclear cells, the amastigotes proliferate in the reticuloendothelial system, especially the spleen, liver, and bone marrow. This causes the hyperplasia of the mononuclear phagocytic system (MPS), causing massive splenomegaly, hepatomegaly, and lymphadenopathy. In addition, the splenic sequestration of the increasing protozoal burden leads to ineffective hematopoiesis. This conundrum leads to changes in bone marrow cell production and hence peripheral cytopenias. Hepatic injury can ensue directly by the protozoa or indirectly by the immune response to the parasites. The resultant liver dysfunction can manifest as jaundice, ascites, and deranged coagulation and depicts a poor prognosis [6].

Many times, patients with visceral leishmaniasis (VL) present with various hematological problems prior to receiving the diagnosis of VL. Serological testing should be performed in suspected leishmaniasis, which shows the protozoal burden in the patient’s system. Polymerase chain reaction (PCR) assay is used for sensitive and rapid diagnosis of *Leishmania* species. However, the most sensitive and confirmatory diagnostic test is bone marrow aspirate revealing the amastigotes. Microscopic analysis can show Leishman-Donovan (LD) bodies, which are either extracellular or intracellular amastigotes (within the macrophages). They are small and round, around 2-4 μm in diameter. They have a small, ovoid, flagellar structure containing an indistinct cytoplasm, a round nucleus, and a small rod-shaped kinetoplast. Extracellular amastigotes are the ones that are released from the disrupted cells [1-6].

Treatment options include pentavalent antimonials (including sodium stibogluconate or meglumine antimoniate), amphotericin B deoxycholate, liposomal amphotericin B, pentamidine, lipid forms of miltefosine, and paromomycin. Although pentavalent antimonials are considered the first-line treatment for visceral leishmaniasis, there has been increasing evidence of drug resistance. Amphotericin B deoxycholate is another highly effective treatment in immunocompetent patients; however, its toxicity and cost are a cause of concern. Liposomal amphotericin B is an excellent alternative that is much less toxic and proves cost-effective [2]. The prognosis can be improved by treating the comorbidities as well. In addition, blood transfusions should be done for severe anemia, along with antimicrobials for coinfections, nutritional supplementation, and adequate hydration [7]. With adequate therapy, patients show improvement within a week, and complete hematological recovery usually occurs in 4-6 weeks of treatment [6]. Relapses are rare, and if no relapses are noted within six months of follow-up, the patient is considered treated [7].

Several factors make the diagnosis of visceral leishmaniasis challenging, including the insidious onset, prolonged febrile nature of the disease, signs and symptoms overlapping with the features of malaria and typhoid, and endemic regions lacking advanced health resources. Unfortunately, most of the endemic regions with heavy disease burden are poor; thus, testing can be incredibly challenging given the tricky presentation of the disease. In addition, the signs and symptoms of visceral leishmaniasis can be vague and misleading, requiring extensive laboratory testing to rule out possible differentials. The chronic and hidden nature of the visceral disease also necessitates frequent doctor visits. Bone marrow aspiration is the gold standard for diagnostic and confirmatory purposes, requiring advanced hospital setups with trained specialists, which poses an additional financial challenge [8].

## Conclusions

Visceral leishmaniasis (VL) is a complex infectious pathology with a heavy disease burden, mainly in Asian and African countries. The diagnosis can be challenging due to many reasons, and a failure to properly diagnose the disease can prove fatal. However, a correct and timely diagnosis can drastically improve the prognosis. This article aims to emphasize the importance of keeping visceral leishmaniasis in the differential diagnoses when a patient presents with a lengthy febrile history and hematological abnormalities. However, the poor socioeconomic status of endemic regions and limited healthcare access remains a problem. Therefore, a multisectoral approach is required to devise comprehensive and economic healthcare accessibility plans.
